# Effects of Exenatide and Humalog Mix25 on Fat Distribution, Insulin Sensitivity, and *β*-Cell Function in Normal BMI Patients with Type 2 Diabetes and Visceral Adiposity

**DOI:** 10.1155/2020/9783859

**Published:** 2020-05-26

**Authors:** Xinlei Wang, Xiaoqin Zhao, Yunjuan Gu, Xiaohui Zhu, Tong Yin, Zhuqi Tang, Jin Yuan, Wei Chen, Rong OuYang, Lili Yao, Rongping Zhang, Jie Yuan, Ranran Zhou, Yi Sun, Shiwei Cui

**Affiliations:** Department of Endocrinology and Metabolism, Affiliated Hospital of Nantong University, Nantong 226001, China

## Abstract

In China, most normal BMI (body mass index of ≥18.5 to <25 kg/m^2^) adults with type 2 diabetes (T2DM) exhibit visceral adiposity. This study compared the effects of exenatide and humalog Mix25 on normal BMI patients with T2DM and visceral adiposity. A total of 95 patients were randomized to receive either exenatide or humalog Mix25 treatment for 24 weeks. Subcutaneous adipose tissue (SAT) and visceral adipose tissue (VAT) were quantified by magnetic resonance imaging (MRI) and liver fat content (LFC) by liver proton magnetic resonance spectroscopy (^1^H MRS). Each patient's weight, waist circumference, BMI, blood glucose, insulin sensitivity, pancreatic *β*-cell function, and fibroblast growth factor 21 (FGF-21) levels were measured. Data from 81 patients who completed the study (40 and 41 in the exenatide and humalog Mix25 groups, respectively) were analysed. The change in 2 h plasma blood glucose was greater in the exenatide group (*P* = 0.039). HOMA-IR and MBCI improved significantly after exenatide therapy (*P* < 0.01, *P* = 0.045). VAT and LFC decreased in both groups (*P* < 0.01 for all) but to a greater extent in the exenatide group, while SAT only decreased with exenatide therapy (*P* < 0.01). FGF-21 levels declined more in the exenatide group (*P* < 0.01), but were positively correlated with VAT in the entire cohort before (*r* = 0.244, *P* = 0.043) and after (*r* = 0.290, *P* = 0.016) the intervention. The effects of exenatide on glycaemic metabolism, insulin resistance, pancreatic *β*-cell function, and fat deposition support its administration to normal BMI patients with T2DM and visceral adiposity.

## 1. Introduction

Diabetes and obesity are primary risk factors for cardiovascular disease (CVD), the leading causes of morbidity and mortality worldwide. About 46.4% of Chinese patients with type 2 diabetes mellitus (T2DM) are at a normal body mass index (BMI of ≥18.5 to <25 kg/m^2^) [1]. Comparisons between European and Chinese populations indicate that normal BMI Chinese adults more frequently exhibit abdominal visceral adiposity than European adults do at a given waist circumference (WC) [2, 3]. The extent of visceral adipose tissue (VAT) correlates negatively with insulin sensitivity and positively with the incidences and development of prediabetes, T2DM [4], and cardiovascular diseases [5, 6]. Moreover, a decreased level of glucose transporter 4 in subcutaneous adipose tissue (SAT) correlates with insulin resistance and T2DM [7].

Nonalcoholic fatty liver disease (NAFLD) is a common chronic liver disease, particularly among T2DM patients, with a global prevalence of 25.24% [8]. The relationship between NAFLD and T2DM can be explained by the link between insulin resistance and hyperinsulinemia. This link leads to dyslipidaemia and triglyceride (TG) accumulation in NAFLD or pancreatic *β*-cell dysfunction in T2DM [9]. Furthermore, VAT also correlates with hepatic steatosis, inflammation, and fibrosis [10] as well as with the severity of fatty liver disease [11]. Thus, considering that NAFLD is common among nonobese patients with T2DM [12], treatments that control blood glucose and glycated haemoglobin (HbA1c) levels while also reducing VAT and liver fat content (LFC) are urgently needed.

Glucagon-like peptide-1 receptor agonists (GLP-1RAs), such as exenatide, are novel T2DM treatments that are used worldwide. Clinical studies have demonstrated that GLP-1RAs can effectively control blood glucose, induce weight loss, protect pancreatic *β*-cells, decrease visceral and hepatic fat deposits, and improve overall and hepatic insulin sensitivity in obese patients with T2DM and prediabetes [13, 14]. However, little is known about the effects of GLP-1RAs on fat distribution and CVD risk factors in normal BMI Chinese patients with T2DM and visceral adiposity.

Insulin, including humalog Mix25, is commonly used for glycaemic control in patients with T2DM. A previous study found that short-term intensive insulin therapy improves pancreatic *β*-cell function, insulin resistance, and lipid parameters in patients newly diagnosed with T2DM [15]. Furthermore, studies have reported conflicting results on the effectiveness of insulin therapy to reduce LFC and consequently increase hepatic insulin sensitivity in obese patients with T2DM [16, 17]. Moreover, few studies have evaluated the association between body fat distribution and insulin therapy in normal BMI patients with T2DM.

Fibroblast growth factor 21 (FGF-21), a circulating hormone derived mainly from the liver in humans, is regulated by nutritional and hormonal factors. Its effects on glucose and lipid metabolism are mediated by adipose and liver tissues [18, 19]. Interestingly, FGF-21 acts selectively on its target organs, including the liver and adipose tissue [20]. FGF-21 might modulate the effects of GLP-1RAs on body fat distribution, namely, through decreases in visceral and hepatic fat deposits.

To elucidate the effects of GLP-1RA on body fat distribution in normal BMI Chinese patients with T2DM and visceral adiposity, we investigated and compared the effects of exenatide and humalog Mix25 on glycaemic metabolism, insulin sensitivity and secretion, fat distribution, and FGF-21 levels in this population.

## 2. Materials and Methods

### 2.1. Subjects and Study Design

Patients were enrolled in the study at the Endocrinology department of the Affiliated Hospital of Nantong University between January 2015 and September 2016. This study was conducted in accordance with the ethical guidelines set forth by the Declaration of Helsinki. The protocol was approved by the Ethics Committee of the Affiliated Hospital of Nantong University (approval number 2015-K002-D01). All patients provided signed informed consent to participate in the study. All study procedures and visits were conducted in the Endocrinology Internal Medicine Laboratory of the Affiliated Hospital of Nantong University. The clinical trial is registered with Chinese Clinical Trial Registry (ChiCTR-IPR-14005568).

### 2.2. Inclusion and Exclusion Criteria

We enrolled T2DM patients who had received a stable dose of any oral antidiabetes drug (except for thiazolidinediones and dipeptidyl peptidase-4 inhibitors) for at least 3 months, an HbA1c level of ≥7.0% to <10.0% at screening or within 4 weeks before screening, a BMI of ≥18.5 to <25 kg/m^2^, and a WC of >85 cm for male or >80 cm for female subjects, respectively.

Subjects were excluded from the study if they met any of the following criteria: (1) current pregnancy, lactation, or child-bearing potential (female subjects); (2) diagnosis or a history of type 1 diabetes mellitus or secondary forms of diabetes; (3) acute metabolic complications of diabetes; (4) treatment with glucocorticoids; (5) a triglyceride level > 4.5 mmol/L; (6) clinically acute or chronic liver disease; (7) moderate/severe renal impairment or end-stage renal disease; (8) significant history of cardiovascular disease; (9) history of chronic pancreatitis, idiopathic acute pancreatitis or gastrointestinal disease and acute or chronic thyroid diseases; (10) diagnosis and/or treatment of malignancy within the past 5 years; (11) history of organ transplant or acquired immunodeficiency syndrome; and (12) history of alcohol abuse or illegal drug abuse within the past 12 months.

### 2.3. Randomization and Administration

Eligible patients were randomized in a 1 : 1 ratio to receive exenatide or humalog Mix25. Based on the order of inclusion in the study, subjects were assigned a random number and then were assigned to one of the two groups. Exenatide (5 or 10 *μ*g/dose, 60 doses, 1.2 mL/filled or 2.4 mL/filled pen) was from AstraZeneca. A 5 *μ*g dose was injected subcutaneously twice daily for 4 weeks, after which a 10 *μ*g dose was injected subcutaneously twice daily for 20 additional weeks. Humalog Mix25 (3 mL pen and kwikpen (prefilled)) was from Lilly. Humalog Mix25 was injected subcutaneously before morning and evening meals for 24 weeks. The patients were contacted once weekly by investigators to discuss glycaemic control. The starting doses of humalog Mix25 were 0.4 IU/kg per day (twice daily) and were then gradually adjusted for target glucose values (fasting plasma glucose (FPG) < 7.0 mmol/L and 2 h plasma blood glucose (2hPBG) < 10.0 mmol/L) by the investigators.

### 2.4. Study Visits and Outcome

Eligible participants underwent a 1-week preintervention screening period (period A) and a 24-week treatment period (period B). During period A, informed consent, demographic data, and medical histories were collected, and the patients underwent physical (height, weight, blood pressure, and waist circumference) and laboratory examinations. Subsequently, the patients were randomly assigned to one of two antihyperglycaemic therapy groups: exenatide or humalog Mix25. All patients underwent an oral glucose tolerance test (OGTT), insulin and c-peptide release tests, magnetic resonance imaging (MRI) to measure the extent of VAT and SAT, and proton magnetic resonance spectroscopy (^1^H MRS) to measure the preintervention LFC.

During period B, study investigators visited patients every week during the first 2 weeks, then every 2 weeks during the following 6 weeks, and every 4 weeks thereafter. At each visit, patients underwent a physical examination and received diabetic education. The diabetic education was taught to each patient in a face-to-face interaction, including how to take food, how to exercise, the control target of blood glucose, blood pressure, lipid, weight, and waist circumference. At each visit, based on the information about diet and exercise and the recorded indices, such as blood glucose, blood pressure, weight, and waist circumference, the individual diabetic education would be taught again. FPG, 2hPBG, and adverse events were recorded, and the doses of insulin and concurrent medications were adjusted by the investigators. At the end of the study, all patients underwent a physical and laboratory examination, including OGTT, insulin and c-peptide release testing, MRI to determine the VAT and SAT, and ^1^H MRS to determine the LFC.

### 2.5. Anthropometric Measurements

Height and weight were measured before the OGTT. Height without shoes was measured to the nearest 0.1 cm by using a stadiometer. Body weight was measured with the lightest clothing to the nearest 0.1 kg by an electronic weighting scale (Tanita TBF-300, Japan). Waist circumference was measured midway between the lowest rib and the superior border of the iliac crest with an inelastic anthropometric tape at the end of normal expiration to the nearest 0.1 cm. Blood pressure was measured by the electronic sphygmomanometer (Omron®, Omron Healthcare, IL, USA). BMI was calculated by dividing weight by the square of height.

### 2.6. OGTT, Insulin, and C-Peptide Release Tests

Subjects reported to the Endocrine Laboratory at 7:00 AM after a 10- to 12-hour overnight fast with no use of the investigated product on the day of the visit. OGTTs were conducted using a 75 g glucose load. Blood samples for glucose, insulin, and C-peptide measurements were collected at baseline and after 30, 60, 90, and 120 minutes from an antecubital vein via a small polyethylene catheter. Plasma glucose levels were measured via the glucose oxidative method (Siemens ADVIA® 2400, Munich, Germany). Insulin and c-peptide levels were measured using chemiluminescent methods (Roche Cobas E411 Analyser, Basel, Switzerland).

### 2.7. Calculation of Insulin Sensitivity, Resistance, Secretion, and Disposition Indices

The presence and extent of insulin resistance were determined using the homeostatic model assessment of insulin resistance (HOMA-IR), as shown in

(1) $$\begin{array}{c} \text{HOMA}‐\text{IR}=\frac{\text{fasting glucose }\left(\text{mmol}/\text{L}\right)\times \text{fasting insulin }\left(\text{mIU}/\text{L}\right)}{22.5}. \end{array}$$

Pancreatic *β*-cell function was determined using the homeostasis model assessment of *β*-cell function (HOMA-*β*), as shown in

(2) $$\begin{array}{c} \text{HOMA}‐\beta =\frac{\left[20\times \text{fasting insulin }\left(\text{mIU}/\text{L}\right)\right]}{\left[\text{fasting glucose }\left(\text{mmol}/\text{L}\right)-3.5\right]}. \end{array}$$

Pancreatic *β*-cell function was also assessed using the Insulinogenic Index (IGI) as shown in

(3) $$\begin{array}{c} \text{IGI}=\frac{\Delta \text{fasting insulin at }30\;\min}{\Delta \text{blood glucose at }30\;\min}. \end{array}$$

The Matsuda index of insulin sensitivity and the MBCI index of insulin secretion were calculated as previously described [21, 22]. The disposition indices were calculated, as shown in Equation (4), to evaluate the relationship between insulin sensitivity and pancreatic *β*-cell function.

(4) $$\begin{array}{c} \text{HOMA}‐\text{IS and HOMA}‐\beta \text{ disposition indices}=\text{HOMA}‐\text{IS}\times \text{HOMA}‐\beta ,\\ \text{Matsuda MBCI disposition indices}=\text{Matsuda}\times \text{MBCI}. \end{array}$$

### 2.8. Fat Tissue Area Distribution

MRI (1.5T HDxt MRI system; GE Healthcare, Milwaukee, WI, USA) with standard array coils was used to measure the VAT and SAT in patients while they were in the supine position. Breath hold fast imaging, with steady-state precession images, was localized to the L4–L5 intervertebral discs. VAT and SAT were defined using the 4 slices exhibiting the best disc alignment and analysed using the Slice Omatic 5.0 software package (Escape Medical Viewer V 3.2). A spline curve was fitted to measure VAT and SAT on the border of the subcutaneous and visceral regions. Nonfat regions within the visceral region were also outlined and subtracted.

### 2.9. Liver Fat Content

LFC was measured using ^1^H MRS. MRI of the liver and in vivo single-voxel MRS were performed using an MRI scanner (GE 1.5T HDxt MRI system) equipped with an 8-channel phase coil. Anatomical T1-weighted spin-echo MR images were localized at the posterior liver lobe, positioned to avoid visible vascular structures. The H_2_O and lipid signal amplitudes were used to calculate the relative LFC as shown in Equation (5) [23].

(5) $$\begin{array}{c} \text{Intrahepatic lipid }\left(\%\right)=\left[\frac{\text{lipid}}{\left(\text{lipid}+{\text{H}}_{2}\text{O}\right)}\right]\times 100. \end{array}$$

### 2.10. Biochemical Measurements

Venous blood samples, which were collected for biochemical measurements at baseline and specified visits, were frozen at -20°C. Blood lipids, liver, and kidney function parameters were measured using enzymatic methods (Siemens ADVIA® 2400). High-performance liquid chromatography (BIO-RAD, VARIANT™ II, Hercules, CA, USA) was used to determine HbA1c, consistent with National HbA1c Standardization Program recommendations. The inter- and intra-assay variations were both <5%. FGF-21 was measured using an enzyme-linked immunosorbent assay (Human FGF-21 SimpleStep ELISA® Kit, Abcam, Cambridge, UK).

### 2.11. Statistical Analysis

Normally distributed measurement data are presented as the means and standard deviations (mean ± SD); the *t*-test of independent samples and *t*-test of paired samples were used for comparisons between two groups and of data collected before and after interventions, respectively. For nonnormally distributed data, the nonparametric Wilcoxon test and symbol rank test were used for comparisons between the two groups and between pre- and postintervention data, respectively, and the results were presented as medians and quartiles.

Continuous data are described as frequencies and rates. The chi-square test was used for statistical analysis when the overall frequency exceeded 40; otherwise, the Fisher exact probability method was used. Differences between the two groups were compared using a mixed effects model with each test index as a dependent variable, group as the fixed effects, and time as the random effects while controlling for age and sex. The relationships between *Δ*VAT and insulin sensitivity, *Δ*VAT and metabolic indices, and FGF-21 and VAT were assessed using Pearson's correlation analysis. The *P* value < 0.05 was considered statistically significant. SAS 9.3 (SAS Institute Inc., Cary, NC, USA) was used for the statistical analysis.

### 2.12. Results

**Figure 1:**
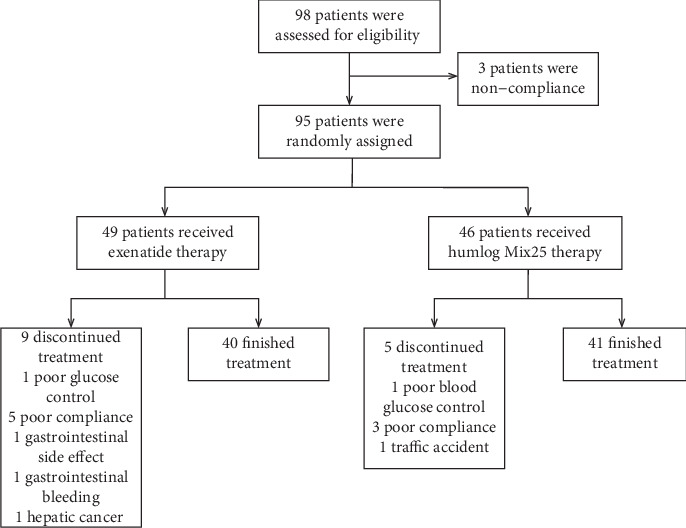
The patient flow diagram.

The baseline characteristics of all 95 patients are shown in Table 1. The two groups differed significantly only in weight, which was greater in the exenatide group (*P* = 0.02), and systolic blood pressure (SBP), which was greater in the insulin group (*P* = 0.03). The final analysis excluded 14 subjects for the following reasons: 2 did not achieve the glucose control target (1 per group); 10 were lost to follow-up, including 8 with poor compliance (exenatide, 5; insulin, 3); 1 was unable to tolerate the gastrointestinal side effects of exenatide; 1 was diagnosed with liver cancer detected by MRI immediately after enrolment; and 2 developed serious adverse events during treatment (gastrointestinal bleeding in 1 patient in the exenatide group; a broken right leg and surgery due to a car accident in 1 patient in the insulin group). Thus, 81 patients (exenatide, 40; insulin, 41) completed the study, and their data were analysed (Figure 1).

#### 2.12.1. Blood Glucose Control

**Figure 2:**
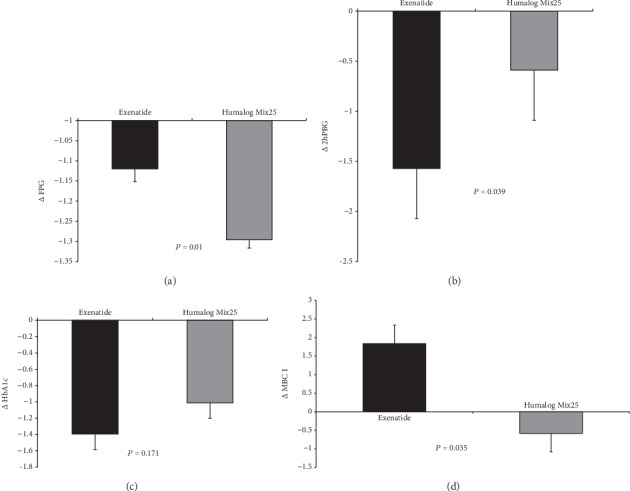
Effects of exenatide or humalog Mix25 on glycaemic metabolism and pancreatic *β*-cell function. Changes in fasting plasma glucose (FPG) (a), 2-hour plasma blood glucose (2hPBG) (b), glycated haemoglobin (HbA1c) (c), and modified *β*-cell index (MBCI) (d) after an intervention with exenatide or humalog Mix25 in normal BMI patients with T2DM and visceral adiposity. *P* values represent comparisons of changes between the two intervention groups.

Both groups exhibited improved glycaemic control. FPG and HbA1c decreased in both groups (*P* < 0.01 for all) (Table 2). The decline in FPG was significantly higher in the humalog Mix25 group (*P* = 0.01), whereas the change in HbA1c was greater in the exenatide group although this difference was not significant (*P* = 0.171) (Figure 2). However, the 2hPBG decreased significantly only in the exenatide group (*P* = 0.03) (Table 2), and this group had a greater *Δ*2hPBG, than the humalog Mix25 group did (*P* = 0.039) (Figure 2).

#### 2.12.2. Insulin Sensitivity, Resistance, Secretion, and Disposition Indices

The HOMA-*β* and Matsuda and IGI indices did not change significantly after treatment with either exenatide or humalog Mix25. However, HOMA-IR deceased and MBCI improved significantly after exenatide therapy (*P* < 0.01, *P* = 0.045) but not after humalog Mix25 treatment (*P* = 0.56) (Table 2). The exenatide group exhibited greater change in MBCI (*P* = 0.035) (Figure 2).

The disposition indices of HOMA‐IS × HOMA‐*β* and Matsuda × MBCI were both higher after exenatide therapy; however, only HOMA‐IS × HOMA‐*β* increased significantly. There were no statistically significant differences in either HOMA‐IS × HOMA‐*β* or Matsuda × MBCI after humalog Mix25 treatment (Table 2).

#### 2.12.3. Loss of Weight and Decrease of WC

Among the 95 patients in the baseline, those in the exenatide group had a higher weight (*P* = 0.02); neither WC nor BMI differed between the groups (*P* = 0.65 and 0.07, respectively) (Table 1). After 24 weeks, the absolute weight lost in each group was significant at -3.55 kg and -1.66 kg in the exenatide and insulin groups, respectively (*P* < 0.01 for all). The WC and BMI also decreased significantly in both groups after the intervention (*P* < 0.01 for all) (Table 2).

#### 2.12.4. Reductions in SAT, VAT, and LFC

**Figure 3:**
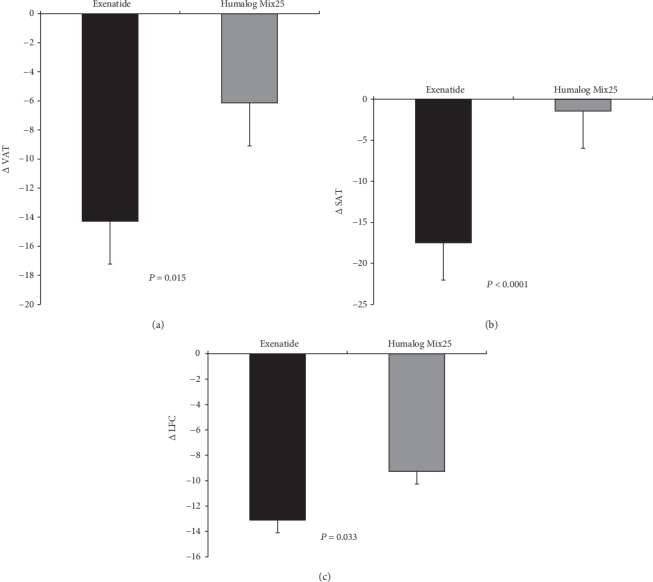
Effects of exenatide or humalog Mix25 on fat distribution and liver fat content (LFC). Changes in visceral adipose tissue (VAT) (a), subcutaneous adipose tissue (SAT) (b), and LFC (c) after exenatide or humalog Mix25 intervention in normal BMI patients with T2DM and visceral adiposity. *P* values represent comparisons of changes between the two intervention groups.

The VAT and LFC also decreased significantly in both groups after treatment (*P* < 0.01 for all) (Table 2), although the absolute decreases were greater in the exenatide group (Figure 3). *Δ*VAT was positively related with *Δ*HbA1c (*r* = 0.268, *P* = 0.018) and negatively correlated with *Δ*Matsuda (*r* = −0.270, *P* = 0.017). SAT decreased significantly after 24 weeks of exenatide treatment (*P* < 0.01) but not after humalog Mix25 treatment (*P* = 0.69) (Table 2).

#### 2.12.5. FGF-21

FGF-21 was only positively related to VAT in the overall cohort at baseline (*r* = 0.244, *P* = 0.043) and after 24 weeks of intervention (*r* = 0.290, *P* = 0.016). Although serum FGF-21 levels decreased in each of the treatment groups, the difference was only statistically significant in the exenatide group (*P* < 0.01) (Table 2). The decrease in FGF-21 in the exenatide group was greater than the humalog Mix25 group (-150.21 ± 215.87 pg/mL *vs.* -36.05 ± 166.33 pg/mL, *P* = 0.016).

## 3. Discussion

Obesity is associated with T2DM, CVD, and visceral adiposity. Previously identified correlations of improved blood glucose control, blood lipid parameters, and increased insulin sensitivity with weight loss in obese patients with T2DM have led to considerable research on weight loss methods [24]. Furthermore, GLP-1RA, a hypoglycaemic agent, can effectively decrease ectopic abdominal adipose tissue, liver fat deposits, and liver enzymes while increasing insulin sensitivity in obese patients with T2DM [14, 25]. However, the effect of GLP-1RA on normal BMI patients with T2DM and visceral adiposity remains unclear. Therefore, this study is aimed at elucidating the effects of a GLP-1RA on the metabolic characteristics, body fat distribution, and CVD risk factors in a final cohort of 81 normal BMI Chinese patients with T2DM and visceral adiposity who completed a 24-week intervention that compared the effects of exenatide (a GLP-1RA) and humalog Mix25 (synthetic insulin). Although both groups exhibited significant decreases in FPG and HbA1c, the 2hPBG only decreased significantly in the exenatide group. Furthermore, the change in 2hPBG was greater with exenatide therapy, whereas a greater decrease in the FPG was observed among patients treated with humalog Mix25. Previous studies have shown that a twice-daily regimen of humalog Mix25 better controlled overnight blood glucose than postprandial glucose in Asian patients and that both FPG and 2hPBG were effectively controlled with exenatide [26, 27]. Therefore, our findings are consistent with those of previous reports.

HOMA-IR is an index that corresponds to fasting glucose and insulin concentrations and highly relates to hepatic insulin resistance. NAFLD is a major cause of hepatic insulin resistance. In our research, *Δ*LFC was greater after exenatide therapy, which may have contributed to the significant change in HOMA-IR after exenatide treatment. MBCI, which presents the overall postprandial pancreatic *β*-cell function in Chinese patients with T2DM [21], revealed that both groups exhibited changes in insulin secretion after the intervention. Glucotoxicity, the toxic effects of persistent and progressive hyperglycaemia, further impairs insulin secretion in T2DM patients. Accordingly, GLP-1RA promotes insulin secretion by inhibiting glucotoxicity [28]. In our study, exenatide was associated with much better glucose control, and the significant improvement in MBCI in the exenatide group may be partly attributable to an alleviation of glucotoxicity. The LIBRA trial, however, demonstrated that both blood glucose control and weight loss contributed to improved pancreatic *β*-cell function observed in subjects with early T2DM during GLP-1RA (liraglutide) treatment [29], and these effects are intertwined [14]. Our findings are consistent with those of the LIBRA trial in that we observed a greater change in body weight, improved insulin secretion (as reflected by the MBCI index), and glycaemic control in the exenatide group. The subjects in our study had been diagnosed with diabetes between 1.5 and 12 years before the trial, leading to speculation that GLP-1RA could also ameliorate pancreatic *β*-cell deterioration in patients with advanced diabetes.

The weight loss associated with GLP-1RA treatment has been attributed to delayed gastric emptying and appetite inhibition via the parasympathetic and/or hypothalamic pathways [30, 31]. The GLP-1 receptor is expressed on both pancreatic and adipose tissues [32]; thus, GLP-1 and GLP-1RA can act directly on adipocytes. In mice, GLP-1RA promotes white adipocyte browning and brown preadipocyte differentiation and increases the utilization of fatty acids and glucose in brown adipocytes [33, 34]. In human adipose tissue, GLP-1 promotes the expression of lipolytic markers and suppresses the expression of adipogenic and lipogenic genes, which have obvious effects on VAT and SAT [35]. In our study, we observed greater decreases in weight, BMI, WC, VAT, and SAT in the exenatide group than the humalog Mix25 group, and our subjects were normal BMI patients with T2DM and visceral adiposity. Given that the effects of GLP-1RA were verified in obese T2DM patients, our findings suggest that GLP-1RA affects the distributions of VAT and SAT, regardless of weight status.

Interestingly, our finding that humalog Mix25 decreased VAT but had a minimal effect on SAT was not consistent with a previous study that reported no effect of an insulin intervention on VAT and SAT [27]. However, Santilli et al. reported that a lifestyle intervention led to reductions in SAT and VAT among patients with T2DM [14]. Therefore, the reduction of VAT in the humalog Mix25 group may be partly attributable to diabetic education at each visit during the 24-week treatment period in our research.

NAFLD is a manifestation of metabolic syndrome, along with obesity, dyslipidaemia, and diabetes [36]. In patients with T2DM, NAFLD increases the risk of diabetic vascular complications and CVD, independent of other known risk factors [37, 38]. Animal studies have shown that GLP-1 analogue therapy improves hepatic insulin sensitivity and decreases steatosis via direct binding to the hepatic GLP-1 receptor [39]. Moreover, human trials have confirmed the ability of GLP-1RA treatment to reverse hepatocyte injury, liver inflammation, and fibrosis [40]. We note that NAFLD is also a frequent comorbidity in patients with nonobese patients with T2DM [12]. Additionally, a preclinical trial of GLP-1RA demonstrated decreased liver inflammation and injury in lean patients with nonalcoholic steatohepatitis [41]. Our cohort of normal BMI T2DM patients can be diagnosed as hepatic steatosis because of high LFC, whereas those treated with exenatide exhibited the largest decreases in this parameter after the 24-week intervention. Therefore, our results reveal the potential benefits of GLP-1RA therapy in patients with normal BMI, T2DM, and NAFLD.

FGF-21 is primarily secreted by the liver into circulation to regulate metabolism. The extracellular protein *β*-klotho, which is expressed in metabolic tissues such as the liver, adipose tissue, and pancreas, binds to FGF-21 to initiate specific signalling to different target tissues [20]. In the liver, FGF-21 reduces hepatic lipid accumulation independent of insulin [42]. Our observation that FGF-21 decreased significantly after treatment with exenatide is consistent with previous research [43]. FGF-21 also has other physiological functions and pharmacological effects. For example, this factor regulates adaptive thermogenesis and enhances energy expenditure and browning in murine adipose tissue [44]. Consistent with these effects, an FGF-21 analogue improved insulin sensitivity and glucose metabolism in humans [45]. In our research, FGF-21 was positively correlated with VAT in the entire cohort before and after intervention. We considered that a high level of FGF-21 at baseline indicated FGF-21 resistance and that the sensitivity of FGF-21 was improved after reduction of VAT for all patients.

Despite our interesting findings, our study was limited by the relatively limited sample size, which might have weakened the statistical power between the two groups. Our results suggest that a prolonged study of a larger group of patients is warranted. Given that we speculated that diabetic education played an important role, a future study should also include a lifestyle intervention group for comparative purposes.

In conclusion, this study of Chinese normal BMI patients with T2DM and visceral adiposity revealed that improvement in 2hPBG, insulin sensitivity, pancreatic *β*-cell function, and SAT were only observed following exenatide therapy. Treatment with this GLP-1RA also led to a greater change in LFC and an improvement in FGF-21 sensitivity. The observed effects of exenatide on glycaemic metabolism, insulin sensitivity, pancreatic *β*-cell function, and fat deposits support the use in normal BMI patients with T2DM and visceral adiposity.

## Figures and Tables

**Table 1:** Baseline characteristics of normal BMI patients with T2DM and visceral adiposity.

	Pre-exenatide therapy (*n* = 49)	Pre-humalog Mix25 therapy (*n* = 46)	*P* value
Sex, female (%)	18 (36.73)	23 (50.00)	0.19
Age (years)	56.10 ± 11.14	60.37 ± 10.83	0.06
Weight (kg)	67.65 ± 9.20	63.87 ± 6.20	0.02
WC (cm)	88.82 ± 5.49	88.37 ± 3.97	0.65
BMI (kg/m^2^)	23.96 ± 1.18	23.50 ± 1.24	0.07
SBP (mmHg)	126.00 ± 14.50	132.54 ± 14.59	0.03
DBP (mmHg)	78.65 ± 7.58	76.74 ± 9.46	0.28
Disease course (years)	8.00 (4.50-14.50)	11.00 (7.00-14.00)	0.08
FPG (mmol/L)	9.44 ± 2.88	9.45 ± 2.11	0.98
2hPBG (mmol/L)	16.40 ± 4.49	17.49 ± 4.08	0.22
FINS (mIU/L)	10.09 ± 3.54	9.86 ± 3.56	0.33
HbA1c (%)	8.53 ± 1.07	8.47 ± 0.92	0.74
TC (mmol/L)	5.10 ± 1.19	5.07 ± 0.95	0.88
TG (mmol/L)	1.58 ± 1.25	1.58 ± 1.01	0.99
AST (IU/L)	22.76 ± 10.38	22.52 ± 5.71	0.89
ALT (IU/L)	26.59 ± 21.29	24.26 ± 11.10	0.50
Creatinine (*μ*mol/L)	58.18 ± 15.35	54.59 ± 12.80	0.22
FGF-21 (pg/mL)	361.78 ± 212.73	283.57 ± 197.53	0.10
SAT (cm^2^)	134.65 ± 47.01	127.87 ± 46.30	0.48
VAT (cm^2^)	83.74 ± 34.52	83.29 ± 38.92	0.95
LFC (%)	23.02 ± 2.92	22.97 ± 3.05	0.41
Smoking, *n* (%)	19 (39.78)	13 (28.26)	0.19
Drinking, *n* (%)	22 (44.90)	24 (52.17)	0.31
Diabetic chronic complications, *n* (%)	2 (4.10)	3 (6.52)	0.47
Sulfonylureas, *n* (%)	18 (36.73)	17 (36.95)	0.58
Biguanides, *n* (%)	27 (55.10)	17 (36.96)	0.06
Glinides, *n* (%)	4 (8.16)	4 (8.69)	0.61
Glucosidase inhibitors, *n* (%)	7 (14.29)	10 (21.74)	0.25

ALT: glutamic pyruvic transaminase; AST: glutamic oxalacetic transaminase; BMI: body mass index; DBP: diastolic blood pressure; FGF-21: fibroblast growth factor 21; FINS: fasting insulin; FPG: fasting plasma glucose; HbA1c: glycated haemoglobin; LFC: liver fat content; SAT: subcutaneous and visceral adipose tissue; SBP: systolic blood pressure; TC: total cholesterol; TG: triglycerides; VAT: visceral adipose tissue; WC: waist circumference; 2hFBG: 2 h plasma blood glucose. Data are expressed as the mean ± standard deviation or median (interquartile range).

**Table 2:** Clinical, biochemical, and imaging parameters of normal BMI patients with T2DM and visceral adiposity before and after exenatide or humalog Mix25 intervention.

Variable	Pre-exenatide	Post-exenatide	*P* value	Pre-humalog Mix25	Post-humalog Mix25	*P* value
	(*n* = 40)	(*n* = 40)		(*n* = 41)	(*n* = 41)	
BMI (kg/m^2^)	23.99 ± 1.2	22.68 ± 1.68	<0.01	23.89 (22.65-24.46)	23.12 (21.48-23.95)	<0.01
Weight (kg)	68.08 ± 9.28	64.53 ± 10.41	<0.01	64 ± 6.39	62.24 ± 7.04	<0.01
WC (cm)	88.83 ± 5.58	84.21 ± 6.57	<0.01	88.39 ± 4.02	84.63 ± 4.76	<0.01
SBP (mmHg)	121.5 (117.5-136)	128 (117-135.5)	0.87	131.73 ± 14.45	128.7 ± 10.6	0.17
DBP (mmHg)	78 (75-84)	76 (70-81.5)	0.10	75.95 ± 9.5	72.2 ± 9.11	0.01
FPG (mmol/L)	9.14 ± 2.41	8.01 ± 2.03	0.01	9.45 ± 2.09	8.17 ± 1.76	<0.01
2hPBG (mmol/L)	15.95 ± 4.28	14.38 ± 3.37	0.039	17.27 ± 4.12	16.91 ± 3.34	0.47
FINS (mIU/L)	9.39 ± 3.13	9.18 ± 2.20	0.56	9.05 ± 2.42	10.28 ± 3.33	0.49
HbA1c (%)	8.43 ± 1.06	7.05 ± 1.04	<0.01	8.41 ± 0.91	7.42 ± 0.83	<0.01
TC (mmol/L)	5.21 ± 1.22	5.03 ± 0.86	0.26	5.01 ± 0.92	4.97 ± 0.79	0.82
TG (mmol/L)	1.35 (0.84-2.12)	1.26 (0.81-1.69)	0.13	1.46 (0.79-2.01)	1.09 (0.77-1.6)	0.01
AST (IU/L)	21 (18-25)	22 (18-27)	0.28	22.61 ± 5.94	22.73 ± 5.39	0.88
ALT (IU/L)	20 (16.5-34.5)	22 (16-30.5)	0.43	22 (18-26)	19 (17-24)	0.13
Creatinine (*μ*mol/L)	57.43 ± 13.63	58.3 ± 13.12	0.52	53 (44-63)	60 (49-66)	<0.01
FGF-21 (pg/mL)	359.64 ± 273.52	209.42 ± 164.22	<0.01	262.46 ± 208.96	226.35 ± 147.13	0.21
SAT (cm^2^)	129.85 ± 43.73	114.18 ± 44.39	<0.01	127.22 ± 48.29	125.7 ± 47.18	0.69
VAT (cm^2^)	80.56 ± 34.26	66.82 ± 30.07	<0.01	76.36 (60.26-96.91)	71 (50.24-88.14)	<0.01
LFC (%)	22.96 ± 3.02	9.83 ± 2.38	<0.01	22.77 ± 3.13	13.44 ± 2.82	<0.01
HOMA-IR	3.99 ± 2.00	2.97 ± 1.79	<0.01	3.48 ± 1.28	3.67 ± 1.59	0.11
HOMA-*β*	42.72 ± 25.63	45.83 ± 21.21	0.63	40.55 ± 19.72	39.88 ± 17.22	0.37
IGI	4.39 ± 1.41	4.92 ± 2.37	0.33	3.77 (1.25-6.08)	4.27 (1.47-6.81)	0.58
Matsuda	5.97 ± 3.96	4.98 ± 2.51	0.15	4.54 (3.15-6.01)	4.58 (3-7.63)	0.78
MBCI	5.44 ± 3.33	7.27 ± 5.04	0.045	4.64 (3.39-6.77)	4.04 (2.83-7.38)	0.56
HOMA-IS∗HOMA-*β*	11.67 ± 5.32	14.44 ± 6.82	0.01	11.85 ± 5.86	12.06 ± 6.69	0.71
Matsuda∗MBCI	22.43 ± 11.61	26.17 ± 13.56	0.37	21.25 ± 11.87	20.57 ± 10.01	0.09

ALT: glutamic pyruvic transaminase; AST: glutamic oxalacetic transaminase; BMI: body mass index; DBP: diastolic blood pressure; FGF-21: fibroblast growth factor 21; FINS: fasting insulin; FPG: fasting plasma glucose; HbA1c: glycated haemoglobin; HOMA-*β*: homeostasis model assessment of *β*-cell function; HOMA-IR: homeostatic model assessment of insulin resistance; LFC: liver fat content; MBCI: modified *β*-cell function; SAT: subcutaneous and visceral adipose tissue; SBP: systolic blood pressure; TC: total cholesterol; TG: triglycerides; VAT: visceral adipose tissue; WC: waist circumference; 2hFBG: 2 h plasma blood glucose. Data are expressed as the mean ± standard deviation or median (interquartile range).

## Data Availability

The datasets used to support the findings of this study are available from the corresponding author upon request.
